# Photoacoustic Spectroscopy Using a Quantum Cascade Laser for Analysis of Ammonia in Water Solutions

**DOI:** 10.1021/acsomega.3c10175

**Published:** 2024-04-17

**Authors:** Apostolos Apostolakis, Guillaume Aoust, Grégory Maisons, Ludovic Laurent, Mauro Fernandes Pereira

**Affiliations:** † Institute of Physics, Czech Academy of Sciences, Na Slovance 2, CZ-18200 Prague, Czech Republic; ‡ MIRSENSE, Nano-INNOV Batiment 863, 8 av de la Vauve, 91120 Palaiseau, France; § Department of Physics, Khalifa University of Science and Technology, 127788 Abu Dhabi, United Arab Emirates

## Abstract

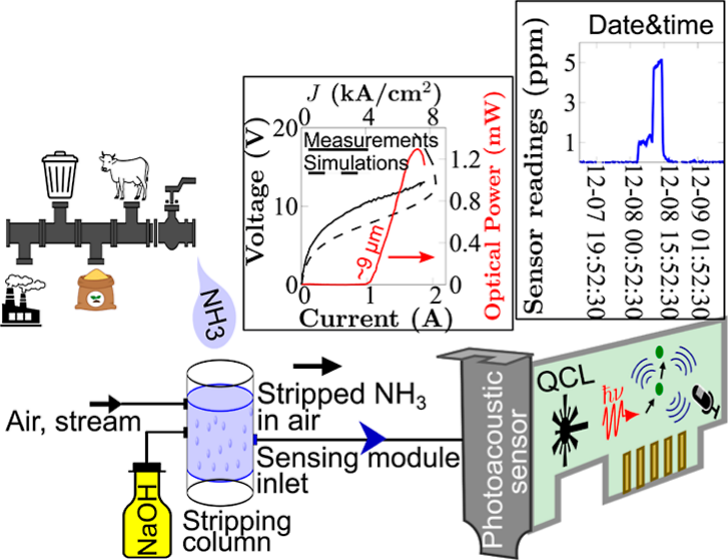

Ammonia (NH_3_) toxicity, stemming from nitrification, can adversely affect aquatic life and influence the taste and odor of drinking water. This underscores the necessity for highly responsive and accurate sensors to continuously monitor NH_3_ levels in water, especially in complex environments, where reliable sensors have been lacking until this point. Herein, we detail the development of a sensor comprising a compact and selective analyzer with low gas consumption and a timely response based on photoacoustic spectroscopy. This, combined with an automated liquid sampling system, enables the precise detection of ammonia traces in water. The sensor system incorporates a state-of-the art quantum cascade laser as the excitation source emitting at 9 μm in resonance with the absorption line of NH_3_ located at 1103.46 cm^–1^. Our instrument demonstrated detection sensitivity at a low ppm level for the ammonia molecule with response times of less than 60 s. For the sampling system, an ammonia stripping solution was designed, resulting in a prompt full measurement cycle (6.35 min). A further evaluation of the sensor within a pilot study showed good reliability and agreement with the reference method for real water samples, confirming the potential of our NH_3_ analyzer for water quality monitoring applications.

## Introduction

Ammonia (NH_3_) is a primary water pollutant present at varying concentrations in both groundwater and surface water. When this nitric waste reaches high levels in water, aquatic organisms find it challenging to discharge the toxicant effectively, leading to toxic buildup. This, in turn, affects the population dynamics of fisheries.^1^ Additionally, ammonia generated in sediments due to nitrification can be toxic to benthic organisms^2^ and surface water biota.^3^

The widespread use of ammonia on farms and in industrial or commercial locations indicates that exposure can occur due to accidental releases.^4,5^ Moreover, the possibility of deliberate events, such as a terrorist attack involving ammonium nitrate,^6^ cannot be ignored. Ammonium nitrate is typically synthesized from nitric acid and household ammonia products.^7^

Continuous water quality monitoring of ammonia is becoming increasingly important for plant operations and the quality control of water utility companies. Taste and odor problems, along with decreased disinfection efficiency, can arise if chlorinated drinking water contains more than 0.2 mg of ammonia per liter.^8^ The drinking water standard recommended by the US National Academy of Sciences,^9^ adopted by many European nations, is 0.5 mg/L (ppm).

As discussed above, uncontrolled releases of ammonia into the environment have a significant impact on human health, ecosystems, fiscal activities, and the climate. This underscores the need for accurate and rapid detection techniques capable of assessing ammonia levels in water over a broad range of concentrations.

While various sensor technologies exist for low ppm detection levels of ammonia in water, ensuring quality assurance and reliability for long-term sensing in complex environments remains a challenge.^10^ Electrochemical sensors, for instance, often face sensitivity issues related to high background ion concentration and the influence of actual field conditions, such as pH, humidity, salinity, and temperature.^11^ In contrast, photoacoustic (PA) sensing schemes incorporating laser sources have shown robust sensitivity under diverse ambient air conditions.^12−14^ Despite the general reliability of spectrophotometry, which relies on the Berthelot reaction,^15^ it still suffers from long analysis times for ammonia nitrogen analysis.^10^ Fiber-optic sensing, another approach employed for the same purpose, has demonstrated promising detection results but with limited repeatability.^16,17^ Additionally, fluorescence-based detection methods face challenges such as reagent optimization and the complexity of the sample matrix.^10,18,19^ Before proceeding further, a brief comment on complementary Terahertz (THz) detection technologies is due. Substances such as NH_3_ and deuterated ammonia (NH_2_D) have several absorption signatures in the THz range (100 GHz to 10 THz). Superlattice multipliers have been recently used to study nitriles in urine as potential markers for kidney damage after chemotherapy.^20^ These type of devices take advantage of nonlinear processes^21^ by acting as frequency multipliers^22^ of electromagnetic waves. While in the present work, NH_3_ is the target, in ref (20), the NH_3_ resonances were so strong in a large spectral range, from to 140 to 791 GHz, that they needed to be avoided. Recent advances in spectroscopy technologies in the mid-infrared (MIR) region^23,24^ and near-infrared (NIR) region^25^ have been exploited to enhance the performances of online water quality monitoring methods. Both approaches can reveal significant details about the molecular-level understanding and chemical properties of the water sample under study. However, spectra in the MIR region provide more specific information about the fundamental molecular vibrations (e.g., stretching, bending, scissoring, wagging), whereas absorption in the NIR region stems either from combination or overtones of fundamental vibrations.^26^ Quantum cascade lasers (QCLs) operate in pulsed or continuous wave mode, at room temperature, with high output power and efficiency for MIR devices that have already demonstrated important applications in the field of trace gas detection using PA spectroscopy,^27,28^ imaging,^29^ and recently for molecules’ detection in aquatic solutions. Current paradigms involving QCL-based spectrometers suitable for water sensing include a thin-film waveguide flow cell accessory coupled to a broadly tunable QCL (between 10.52 and 11.23 μm) facilitating low ppm detection (∼5 ppm) of chlorinate hydrocarbons traces in water^30^ and a chip-based evanescent wave sensing platform (QCL light emitted between 6.5 and 7.5 μm) allowing the detection of aqueous toluene in a limit of 7 ppm.^31^

In spite of the certain progress in the laser-based water sensing^23,30−32^ and gas sensor applications^33^ for water quality monitoring, automated and reliable systems using these sensing techniques for detection of ammonia in water are far from being achieved.^34^

In this work, we present a sensor based on a PA MIR spectrometer trace gas analyzer and an automated ammonia stripping method to determine the levels of ammonia–nitrogen concentration in water samples. A PA gas sensor device is a device capable of analyzing gas based on the PA effect.^5,14^ Other intriguing methodologies for gas detection encompass triboelectric generators and sensors employing chemoresistors.^35−38^ In our system, the PA gas sensor device incorporates a QCL emitter module generating MIR light pulses to be absorbed by a gas containing ammonia molecules, which can be stripped from the water phase into the gas phase by a specially designed stripping system. The nonradiative relaxation of the excited molecules results in an acoustic wave that is detected by a pressure-sensitive module and thereafter generate a corresponding sensor signal. Detection limits are established for both the gas analyzer and the overall performance of the system for tracing ammonia molecules in water. Moreover, the ammonia analyzer underwent sensitivity and stability tests to optimize the total measuring and sampling run cycle under laboratory conditions, in addition to near real-time quantitative analyses of ammonia under field conditions in realistic test cases. The results obtained indicate the potential of the sensor for near real-time water monitoring involved in the control of wastewater plants and environmental applications.

## Experimental Section

### Chemicals and Preparation of Liquid Samples

A standard solution of each water sample was prepared by following the standards.^39^ Specifically, ammonia stock solution (1000 ppm) was prepared by dissolving accurately 3.819 g of anhydrous ammonium chloride in deionized (DI) water and diluting to 1000 mL in a volumetric flask, whereas the ammonia working standards (e.g., 10 ppm) were prepared by diluting 10 mL of ammonia stock solution to 1 L of DI water in a volumetric flask. The reagent solution (0.8 mL of 3% w/w NaOH per measurement cycle) ensured the maximum removal efficiency of ammonia.

### Integrated Sampling Operation of the NH_3_ Analyzer

**Figure 1:**
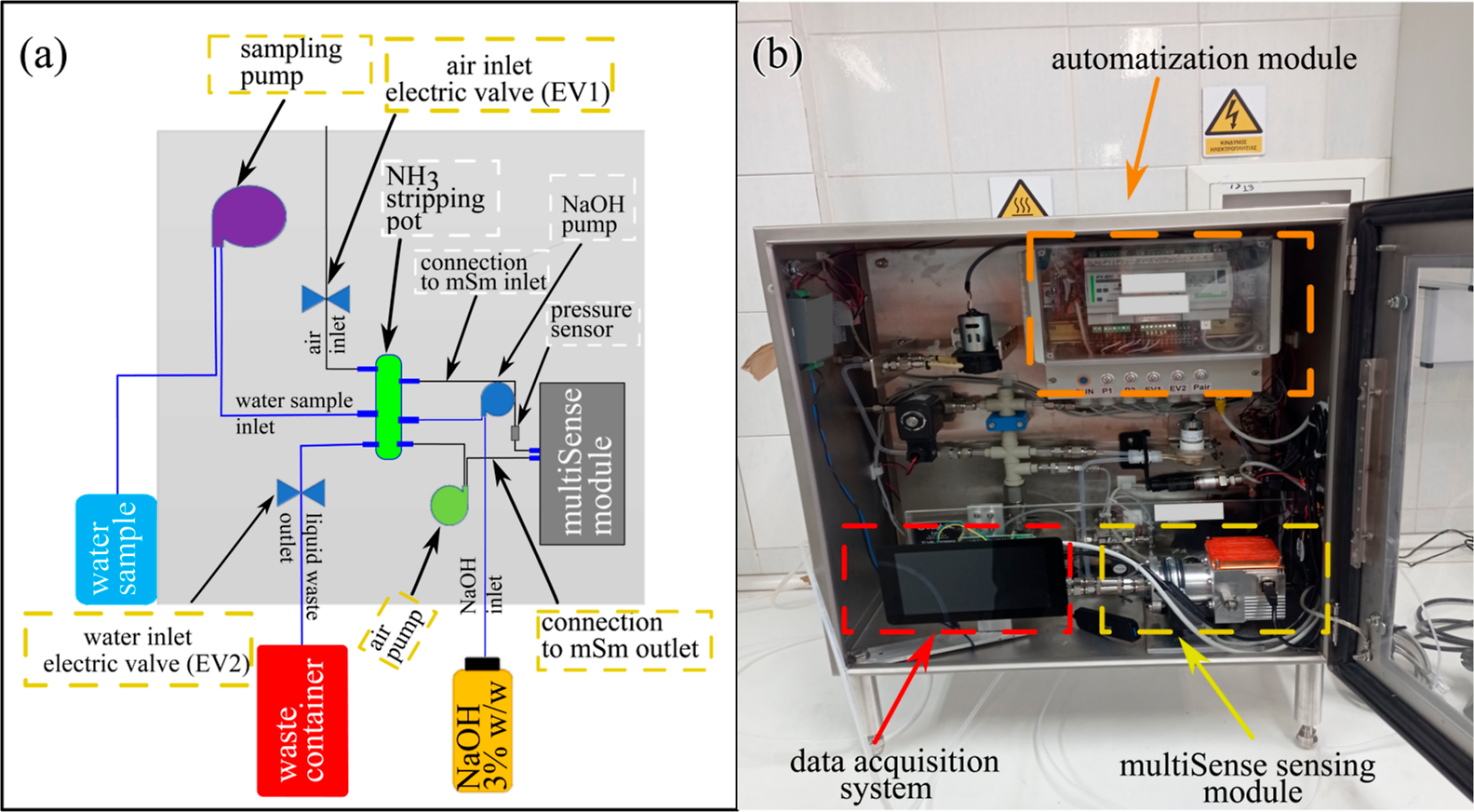
(a) Schematic setup of the gas and water sampling lines and flow in the ammonia analyzer. (b) Picture of the sensor including the multiSense sensing module, the data acquisition system, and the automatization box.

Figure 1a shows the schematic diagram of the sampling lines in the ammonia analyzer, which allows automated water sampling. The assembly of the ammonia stripping/liquid sampling subsystem was provided by Swagelok^40^ following our design and technical specifications. After the reagent and water samples were prepared as discussed in the previous subsection, they were loaded into the corresponding containers. First, the EV1 valve opens, allowing air flow while the air-pump (diaphragm pump KNF-NMP05) is powered on in order to bring fresh air at a mean rate of 450 mL/min from outside the enclosure into the sampling circuit. Thereafter, the water sample is injected into the stripping column using a peristaltic sampling pump at a flow rate of 220 mL/min, and subsequently, a different peristaltic pump is turned on to add NaOH solution at a flow rate of 28 mL/min into the same reservoir. Thereafter, EV1 and EV2 valves are closed, whereas the air pump is reactivated to flush air within the column, igniting the stripping process of NH_3_ and thereafter the tail gas, a mixture of air and ammonia, is released through the top of the stripping column and then absorbed by the PA gas-sensing module. Finally, reopening the EV1 and EV2 valves results in transferring the analyzed water sample into the waste container and therefore completing the full-sampling cycle. To fill the stripping column with water sample, a silicone tubing of 1.5 m length × 6 mm outside diameter (O.D.) was connected to the sampling pump using a tubing adapter. The same type of silicone tubing was used to connect the outlet of the stripping pot to the waste container, whereas a PFA tubing (1.5 m length × 3.18 mm O.D.) was connected to the peristaltic pump to inject the reagent solution to the stripping pot. The remaining portion of the liquid sampling system was designed using PFA tubing, renowned for its low coefficient of friction and antistick properties.^41,42^ The PA cell and sampling lines of the gas sensor, constructed from stainless steel, aid in suppressing external noise and ensuring minimal surface absorption of the gas being investigated.^43^

### Spectral Range Selection Process

**Figure 2:**
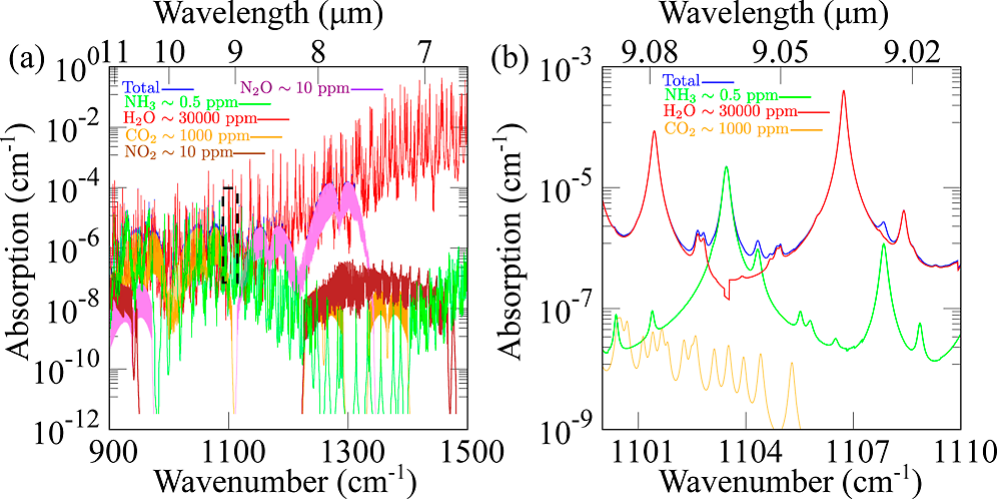
(a) Spectroscopic simulations of a gaseous matrix containing ammonia gaseous within the MIR region. The dashed frame depicts the specific selection of the spectral window for tuning the wavelength of the QCL. (b) Recommended absorption band for ammonia detection in the spectral region from 1100 to 1110 cm^–1^. The temperature is considered *T* = 333 K, whereas the pressure is *P* = 1 atm.

The measurement method relies on stripping of free ammonia (Figure 1a) from water into the gas phase, where the spectrometer module (Figure 1b) can effectively detect the target molecules. Thus, it is critical to identify the wavelength corresponding to the light of the laser source, which can be absorbed by a specific gaseous specie. The simulated absorption spectra in Figure 2a of the gas matrix including simple gas molecules such as water (H_2_O), carbon dioxide (CO_2_), nitrogen dioxide (NO_2_), and the targeted gaseous ammonia were performed using the parameters from the HITRAN database.^44^ Therefore, the carrier-gas stream involved in the stripping process is considered of typical composition as found in moist air.^45^ To ensure that the ammonia-enriched gas matrix can be modeled realistically, the following features were assumed: (i) the gas molecule of interest is present at a significantly low concentration (∼0.5 ppm), (ii) the other interfering species, which are carried by the carrier gas, are present at their maximum potential concentration, e.g., levels of CO_2_ and H_2_O at ∼1000 and 30,000 ppm, respectively. In our case, the N–H bond wagging vibration mode in the proximity of 8.78 μm^46^ has been chosen due to strong fundamental absorption of the ammonia molecule in the spectral region 8.6–9.1 μm, which can be covered by the lasing technology included in the MIR sensing module, whereas the other MIR regions reveal pronounced overlaps with absorption bands of H_2_O, and therefore, they are not suitable for detection of stripped ammonia. The choice of this spectral window was also suggested by Owen et al.^47^ and has been more recently employed in ref (48). This consideration led us to a more specific selection of the wavelength region (depicted by the black-dashed frame in Figure 2a), where a robust ammonia absorption feature aligns with minimal water absorption and other minor interferences, as further illustrated in Figure 2b. The zoomed-in plot of the absorption spectra reveals a potential peak interferent attributed to water vapor. To mitigate this interference, we opt for a wavelength near 1103.46 cm^–1^, corresponding to a maximum absorption intensity α_*G*, max_ = 2.23 × 10^–5^ cm^–1^, where the absorption lines of CO_2_ and H_2_O are notably weaker. Further information on the wavelength selection of the QCL source is provided in the Supporting Information, specifically in Figures S1 and S2.

### PA Spectrometer Design

The MIR spectrometer module (multiSense) developed by mirSense company^49^ was employed, which consists of a QCL laser and a PA cell allowing detection of gas molecules at sub-ppm limit. In detail, the multiSense module (mSm) has been fabricated as a semi “stand-alone” solution (170 mm × 110 mm × 110 mm) within a rack of *W*550 mm × *H*575 mm × *D*300 mm, where adequate sampling conditions are assured, i.e., heated sample lines, flow control, pressure measurement, and software control (Figure 1b). The control of the entire system (mSm, stripping module, and pressure sensors) was managed by a tablet running Windows 7. This tablet hosted the necessary software to establish communication with the aqua3S data collection platform and retrieve the data. Additionally, a complementary software was developed for the aqua3S project to generate a JSON file, which facilitates data communication with the aqua3S platform.^50^

**Figure 3:**
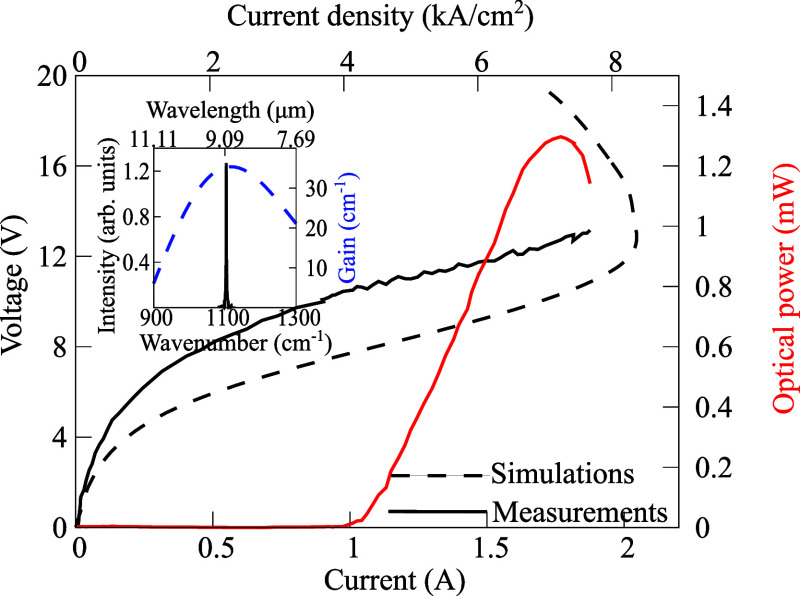
Power-current–voltage characteristics of a QCL structure InGaAs/AlInAs at room temperature. The dashed and solid lines depict the simulated curve calculated with the NEGF approach and the measured characteristics, respectively. The inset illustrates the obtained optical spectra in comparison to the numerical calculations of the optical gain.

The gas mixture retrieved by the stripping process is considered an air mixture, as indicated in the previous section with a temperature ranging between 263 and 323 K and a typical pressure of 1 atm. Thus, the measurement gas cell within mSm is regulated at 333 K, well beyond the upper limit of 323 K, to make certain that the cell temperature will not drift due to an elevated temperature. The mSm employs the conventional photoacoustic spectroscopy (PAS) technique using commercial microphones. The QCL operates in pulsed mode, allowing us to reduce the heat produced within the system.^51^ The light radiation has a wavelength that varies periodically about the central wavelength corresponding to the absorption peak of NH_3_, a value specifically suitable for the excitation of a gas to be detected. In particular, the QCL source emits radiation close to the selected absorption line (*k* ∼ *k*_NH_3__) illustrated in Figure 1b, while operating in a quasi-continuous wave regime at room temperature. The design of the QCL structure is based on the InP wafer, whereas the repeats of AlInAs/GaInAs ternary layers correspond a double-phonon resonant design with four quantum well active regions optimized at λ ∼ 9 μm. The simulated current–voltage characteristic based on a nonequilibrium Green’s functions (NEGF) method^52−55^ agrees well with the experimental one shown in Figure 3. Our computations rely on the stationary state established under operational conditions of the device, derived from self-consistent solutions of quantum kinetic equations governed by nonequilibrium Keldysh Green’s functions. The mechanisms of electron–electron, electron–phonon, impurity scattering, and interface roughness scattering were determined through their respective self-energies.^53,54,56,57^ The voltage difference of experiment to simulation is attributed to the nonoptimized input parameters of the scattering self-energies of every implemented scattering mechanism. The gain profile of the QCL was determined using our comprehensive NEGF model,^57,58^ applying a bias of 280 mV per period. Subsequent comparison with experimentally obtained spectra of the laser device (inset in Figure 3) verifies our findings, indicating a consistent match between the calculated peak gain frequency and the measured frequency of the QCL device. The corresponding optical spectrum of the laser was obtained with 300 ns long pulses and with a duty cycle of 3% and a temperature of 293 K. These operational parameters were used for the electrical characterization of the QCL structure, and they do not represent the laser working parameters during the process of the PA detection. To achieve a single frequency emission, a Bragg grating was used to select the wavelength. The period of grating allows the tuning of the emitted wavelength over the gain of the active region. Furthermore, the laser is a double trench design and has been epi-up mounted on the aluminum nitride submount.

To determine the minimum optical power *P*_*l*_ required by the emission of the fabricated QCL, we started by assuming a normalized noise equivalent absorption (NNEA) coefficient of 2 × 10^–8^ W cm^–1^ Hz^–1/2^, which is a known metric of the PA detector’s sensitivity^12,59^ and a detection bandwidth of Δ*f* = 1.7 × 10^–2^ Hz (inversely proportional to detection time Δ*T* = 60 s). The minimum detectable absorption is commonly defined as .^60^ Thus, given the selection of the absorption peak α_*G*, max_ illustrated in Figure 2, the required optical power is  mW and thereafter, we adjusted accordingly the duty cycle to match this optical power. The overall parameter adjustments of the PA spectrometer resulted in a detection limit (3σ) of 0.18 ppm for gas-NH_3_ concentration in 60 s averaging.

In addition, the linearity of measurement within a 0–500 ppm range was demonstrated. A resolution below 0.1 ppm was also shown for gas phase measurements with the mSm module. For further details of the mSm module operation, refer to Figures S3–S5 of the Supporting Information.

The features of the PA spectrometer as presented here indicate its potential for determination of traces of ammonia in water by means of NH_3_ stripping, which allow for the separation of free-ammonia into the gas phase.

### Ammonia Stripping Process

The ammonia stripping process is a stripping method based on the principle of mass transfer.^61,62^ The ammonia stripping/sampling compartment of the analyzer was structured as depicted in Figure 1a, with the stripping column positioned at the center of the rack. Specifically, in this method, water is contacted with air to strip the ammonia gas present in the water. The presence of ammonia in water can be found in two forms, namely, ammonium ions (NH_4_^+^) and ammonia gas (NH_3_). The relative concentrations of ammonia gas and ammonium ions are directly dependent on the pH and temperature of water.

Ammonia nitrogen in water exists in equilibrium between the molecular and ionic form according to the following reaction
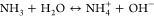
1whereas the dissociation of water is given by the equilibrium reaction
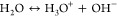
2The ammonia fraction, *f*_NH_3__, determines the concentration ratio between free ammonia [NH_3_] and total ammonia . Typical stripping processes require a sample temperature between 293 and 323 K, whereas pH values range between 10 and 12. This follows from the dependence of free ammonia on the pH and temperature (see Figure S6). Therefore, a basic solution acting as reagent is needed to regulate the pH levels. In our case, a NaOH solution was used, resulting in significantly alkalinized pH ∼ 12 and therefore to an ammonia fraction close to 1. Complementary description of the ammonia stripping process is given in the Supporting Information.

## Results and Discussion

**Figure 4:**
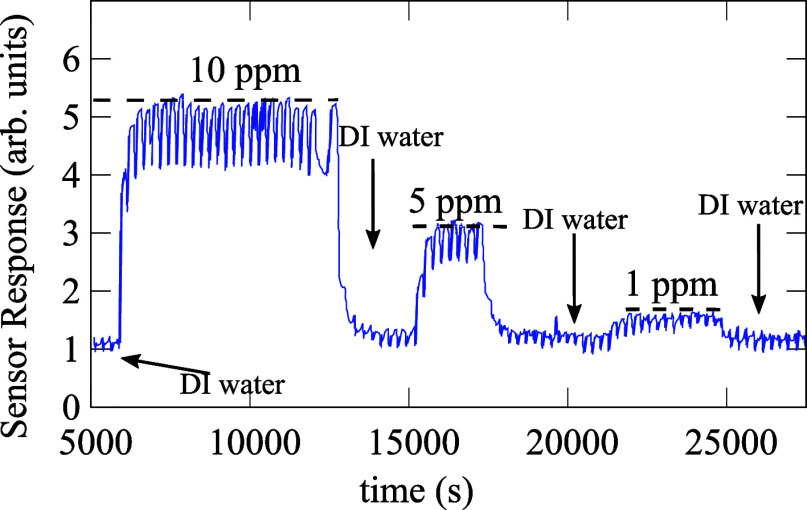
Dynamic response of the sensor to three different concentrations of NH_3_ in water at room temperature. These records are interrupted by deionized water rinse.

**Figure 5:**
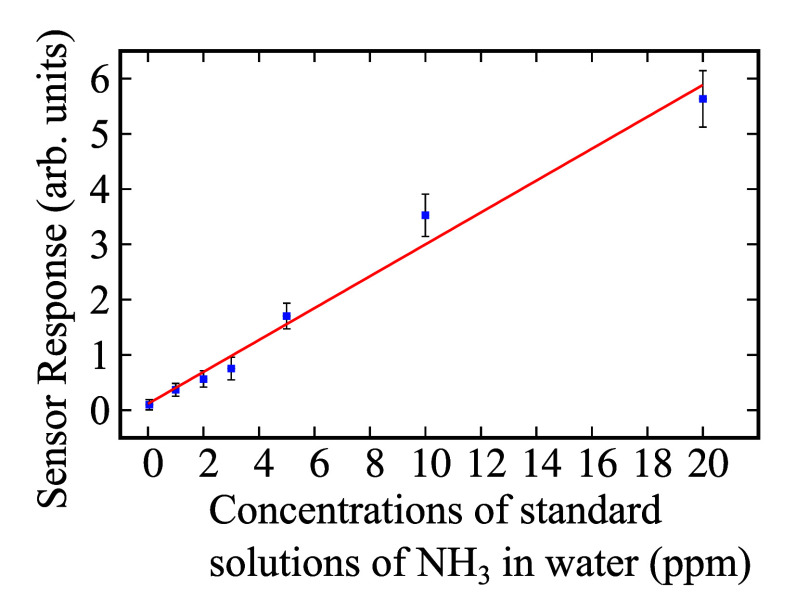
Sensor response (calibration curve) within the range of 1 to 20 ppm of NH_3_ concentration in water at room temperature.

**Figure 6:**
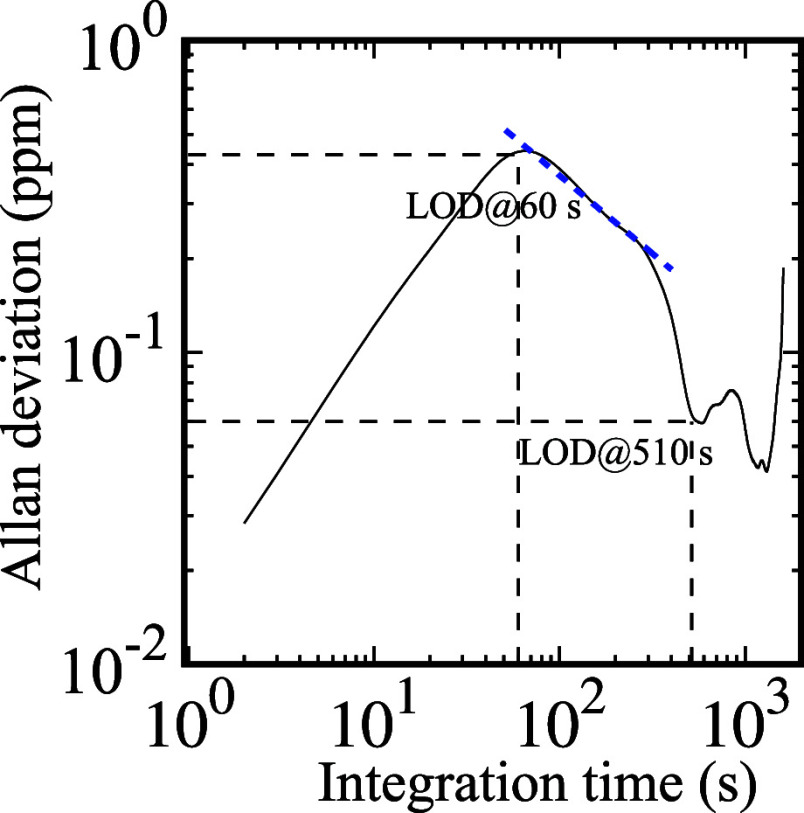
Allan–Werle deviation analysis recorded on 10 ppm of ammonia concentration in water. The blue dashed line indicates a typical dependence  for white noise.

The sensor calibration for the detection of ammonia traces in water was achieved by using standard reference solutions (see chemicals and preparation of liquid samples) starting from a NH_3_ stacking solution of 1000 ppm, which is diluted to obtain reference samples of lower concentrations of ammonia. The monitoring of ammonia is hindered by the sticky nature of its polar molecule^65^ that commonly adheres to inert surfaces. To prevent the attenuation of sticky molecules such as ammonia within the tubes during the sampling process, we carefully stored the samples under refrigeration. On that account, samples above 233 K were avoided in order to suppress the condensation risk in the pipelines. As is well-known, condensation makes NH_3_ to be dissolved in water droplets immediately,^66^ compromising the reliability of the measurement. Figure 4 demonstrates consecutive records with reference samples 10, 5, and 1 ppm of ammonia concentrations. Each oscillation represents a measuring cycle (6.35 min corresponding to 1 s time step) including the sampling time of the water sample of ∼0.4 min, the sampling time of the NaOH reagent solution of ∼0.05 min, and the purge time of ∼0.7 min. These records are intermitted (vertical arrows) by measurements of blank solutions; 2 mL NaOH-DI water samples were used as blank solutions, which indicated that their related memory effects do not affect significantly the consecutive measurements. In turn and as anticipated, the relationship between the reference samples and the sensor response (Figure 5) is linear (Pearson’s *r* = 0.99). Note that by considering a low time constant and 60 s of averaging, we obtain a limit of detection (LOD) of just about 0.4 ppm. By resorting to an Allan–Werle variance method^67,68^ with a time constant of 1 s and a 10 ppm concentration of ammonia in water, the limit of detection may reach down to 0.4 ppm for an integration time of 60 s (Figure 6). With an increase of averaging time, the Allan–Werle plot demonstrates a continuous decrease, revealing a white-noise prevalent behavior (∝*t*^–1/2^) of the sensor response as shown by the blue dashed curve. However, for averaging times of up to approximately 60 s, this trend line is superimposed with a short-term trend, likely attributable to mechanical vibrations induced by the liquid sampling system, which can exhibit behavior akin to an inertial subrange.^68^ In such circumstances, the Allan deviation offers an estimation of measurement precision in terms of a Gaussian standard deviation, which improves with increasing averaging time while maintaining consistent accuracy. Therefore, we identify our theoretical estimation of LOD as moderately accurate, corresponding to the first point along the *t*^–1/2^ trend line. Moreover, Figure 6 suggests that the minimum LOD can be further improved to 0.06 ppm with an integration time of 500 s, whereas a longer integration time clearly indicates the emergence of a long-term drift.

The sensor performance developed in this article is compared with previously demonstrated QCL-based sensor systems suitable for the detection of substances in water and other sensing techniques, with a focus on ammonia detection, as shown in Table 1. The star symbol indicates uncertainty regarding whether the time provided corresponds to the detection time alone or the entire acquisition time, including sampling or other processes, in these works. The optimized detection limit of 0.4 ppm stands out as particularly competitive among other reported detection techniques, while the enhanced detection range and acquisition time further underscore its potential for water quality monitoring.

**Table 1:** Performance Comparison of the Newly Developed PA QCL-Based Sensor System vs Previously Reported Sensing Techniques

detection technique	light source	wavelength (μm)	target molecule	acquisition time	detection range (ppm)	LOD (ppm)	refs.
MCT detector	QCL	10.98	CHC	33 min	5–20	5	(34)
HgCdTe detector	QCL	6.5–7.5	toluene	2 min	7–100	7	(31)
FTIR	IR spectrometer IFS-113v	2.5–25	NH_3_		1–10	1	(34)
fiber-optic	halogen lamp SL201/M		NH_3_	0.2 min^★^	10–50	10	(16)
electrochemical			NH_3_	>8 min		10	(11)
membraneless gas-diffusion			NH_4_^+^	11 h	10–50	2.2	(63)
colorimetric			NH_3_	10 min	10–1020	10	(64)
spectro-photometric			NH_3_	60 min	0.015–12	0.015	(15)
fluorescence			NH_3_	1.7 min^★^	1.8–560	1.8	(18)
photoacoustic	QCL	9	NH_3_	6.35 min	0.4–500	0.4	this work

### PA NH_3_ Analyzer: Pilot Studies

**Figure 7:**
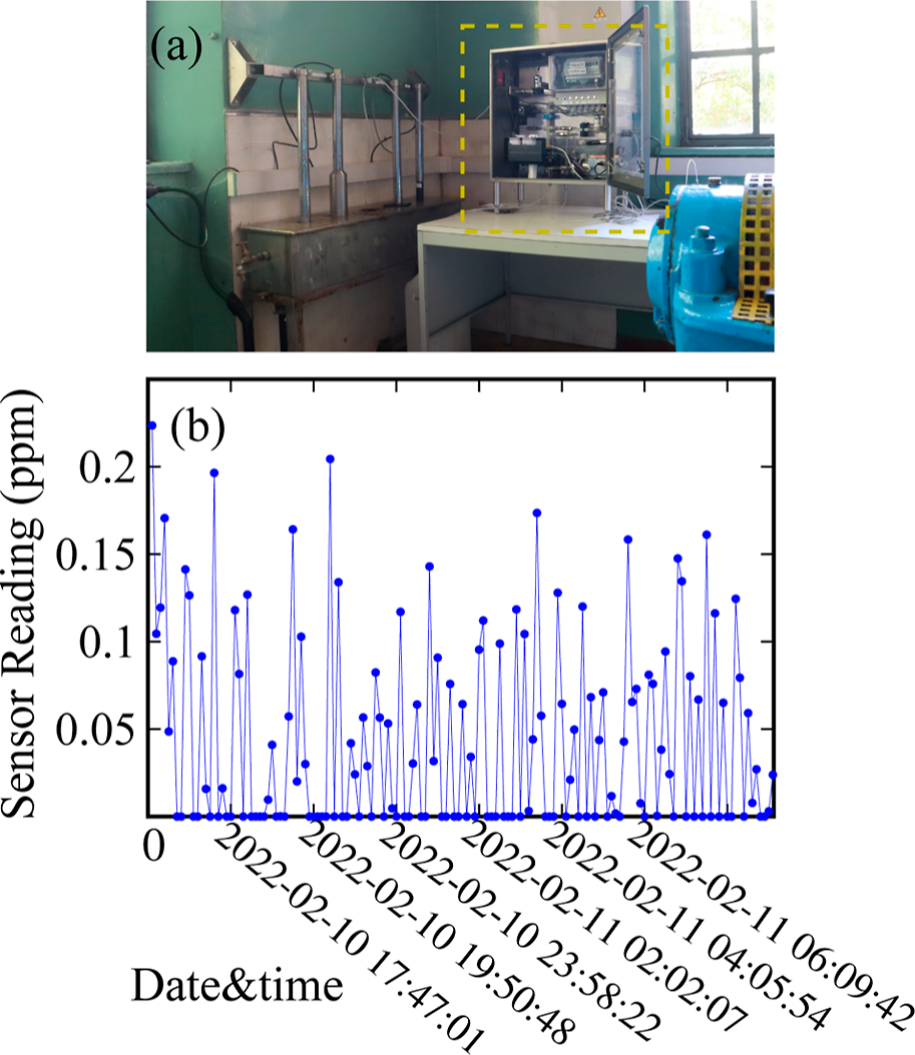
(a) Field test of the sensor (highlighted by the yellow frame), which was deployed in a pumping station during a pilot case study. (b) Sensor reading as a function of time indicating the concentration of ammonia traces within spring water. The original photo in (a) is a photograph courtesy of Gerasimos Antzoulatos from CERTH. Copyright 2024.

The laboratory results indicated that the designed device could effectively detect the presence of ammonia in water. To validate and further develop this innovative technology, the sensor was installed in different pilot cases within the context of H2020 project aqua3S,^69^ which aimed to standardize existing sensor technologies complemented by state-of-the-art detection mechanisms focusing on water safety and security. In particular, the sensor was deployed at Trieste Aqueduct (Randaccio site), Italy, and Quality Control laboratory located at the Thessaloniki Water Treatment plant (TWTP), Greece. In the first pilot case, the sensor was continuously monitoring the water stored in a pumping station (Figure 7a), showing practically no response (Figure 7b), which is consistent with the historical data (<0.05 ppm) of ammonia concentration determined by analytical measurements; the currently estimated LOD of the sensor is above this range. The data observed by end-users (Figure 7b) correspond to a single point determined by the running average of the last 60 s of the measurement time (i.e., the last 60 s of the 5.2 min interval), ensuring enhanced accuracy and better stability of the signal.

**Figure 8:**
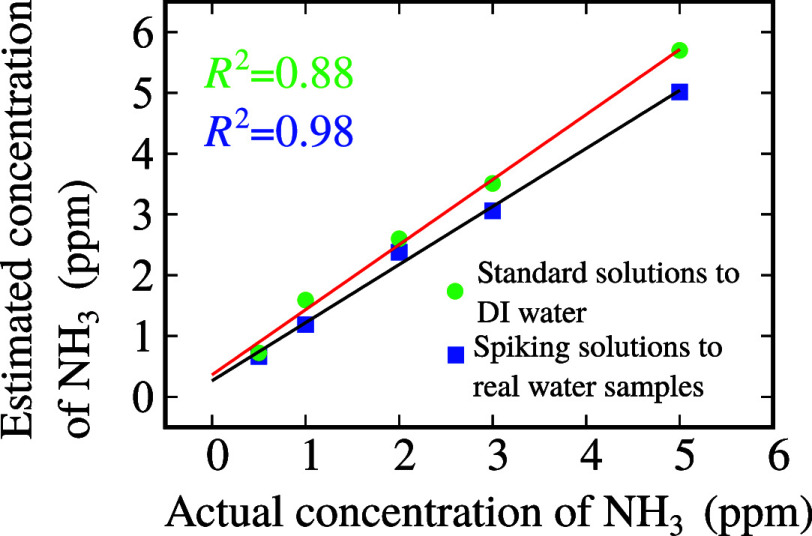
Determination of ammonia concentration in real water samples (black curve) and DI water (red curve).

In the second pilot case, the optimized sensor system, which is equipped with automated sampling, was applied first for determination of NH_3_ in real samples collected from the Δ2 tank of the TWTP, spiking different ammonia concentrations directly into the water samples. Figure 8 displays a good linear response (black curve) of the sensor to real water samples with a linearly dependent coefficient *R*^2^ = 0.98. This result indicated the reliability of the approach with practically no-matrix effects due to formation un-ionized ammonia at high pH values resulting from the use of NaOH-buffer solution. The wide linear range of the sensor comfortably allowed for measurements for ammonia concentration larger than 2 ppm, which were critical for the implementation of the pilot scenario considering an extended ammonia spillage event in the water storage tank of TWTP. The recovery values of ammonia are presented in Table 2, showing a recovery range from 112 to 147.2%. The values in the second column correspond to measurements obtained through other analytical methods,^70^ not the sensing technique employed in this study. These water samples were taken from the same collection batch, spiked with ammonia, and utilized for detection via a PA analyzer. Furthermore, the enhanced recovery rates can be attributed to ongoing conditioning or measuring cycles, which might result in minor memory effects due to residual ammonia molecules released in the course of successive measurements.

**Table 2:** Recovery Study Performed by Adding Standard Solutions of Ammonia to Real Water Samples

NH_3_ added (ppm)	NH_3_ found before spiking (ppm)	expected (ppm)	measured (ppm)	recovery (%)
0.5	0.08	0.58	0.72	124.4
1	0.08	1.08	0.72	147.2
2	0.08	2.08	2.59	124.4
3	0.08	3.08	3.5	113.6
5	0.08	5.08	5.69	112

## Conclusions and Outlook

The work presented here describes the development and characteristics of a sensor system suitable for detection of ammonia traces in water by employing a PA sensing technique. To the best of our knowledge, this is the first demonstration of a QCL-based ammonia detector combined with an automated ammonium ion stripping process. The NH_3_ analyzer demonstrated high sensitivity with a detection limit of 0.4 ppm, which is considerably low when compared with other devices incorporating QCL structures for detection of molecules in water or electrochemical gas sensors integrated with ammonia stripping modules. Near real-time monitoring of ammonia concentration in real water samples was performed in the course of pilot field case studies, and the sensor could detect ammonia in real water samples. As a result, these results outline the potential of gas sensor applications and PA sensing particularly in systematic water quality monitoring. Currently, we are redesigning our water sampling method to allow for the detection of other molecules in water beyond ammonia. In order to extend the feasibility of our approach at part per billion levels, improvements in the water/gas sampling circuits should be considered such as reduction of the noise generated by the air-pump and sampling pump, decrease of the cold points of the instruments in order to avoid water condensation issues, enhancement of the efficiency of the purging steps, redesign of the water reservoir to allow for an increase of ammonia recovery at low concentration levels, and finally, further optimization of the QCL structure and lasing power, which can emit at the characteristic wavelength, which is resonant with the vibrational excitation of ammonia molecules. Future work on the sensor could concentrate also on enhancing long-term stability by employing solutions such as proposed pressure reduction,^71^ implementing reference cell temperature stabilization,^72^ exploring various signal processing algorithms,^73^ and other potential strategies.

## Supporting Information Available

Information with respect to the processes involved in the specifics of the measurement wavelength selection and evaluating the performance of the gas sensor (mSm) response; assessing the performance of the mSm in measuring gaseous ammonia and data on a linear response range; data on the sensor’s response near the limit of detection; and additional information regarding the ammonia stripping process under consideration (PDF)
(ZIP)
