# Supplementary material for: Photoacoustic Spectroscopy Using a Quantum Cascade Laser for Analysis of Ammonia in Water Solutions
Source: ACS Omega. 2024 Apr 17;9(17):19127–35. doi: 10.1021/acsomega.3c10175 (PMC11064027; doi:10.1021/acsomega.3c10175)
Supplement: Supplementary file 2 — ao3c10175_si_002.zip [file ao3c10175_si_002.zip › acsSupportingInformationfinal.pdf]

SUPPORTING INFORMATION

Photo-acoustic spectroscopy using a quantum cascade laser (QCL) for analysis of ammonia in water solutions

Apostolos Apostolakis,*,† Guillaume Aoust,‡ Grégory Maisons,‡ Ludovic Laurent,‡ and Mauro Fernandes Pereira*,¶,†

†*Institute of Physics,Czech Academy of Sciences, Na Slovance 2, CZ-18200, Prague, Czech Republic*

‡*MIRSENSE,Nano-INNOV Batiment 863, 8 av de la Vauve, 91120 Palaiseau, France*

¶*Department of Physics,Khalifa University of Science and Technology, 127788, Abu Dhabi, United Arab Emirates*

E-mail: apostolakis@fzu.cz; mauro.pereira@ku.ac.ae

Section S1

In this section, we complement the information presented in the main manuscript by including additional details on the spectral range selection process. Two figures (S.1-2) are provided to illustrate the specifics of the measurement wavelength selection.

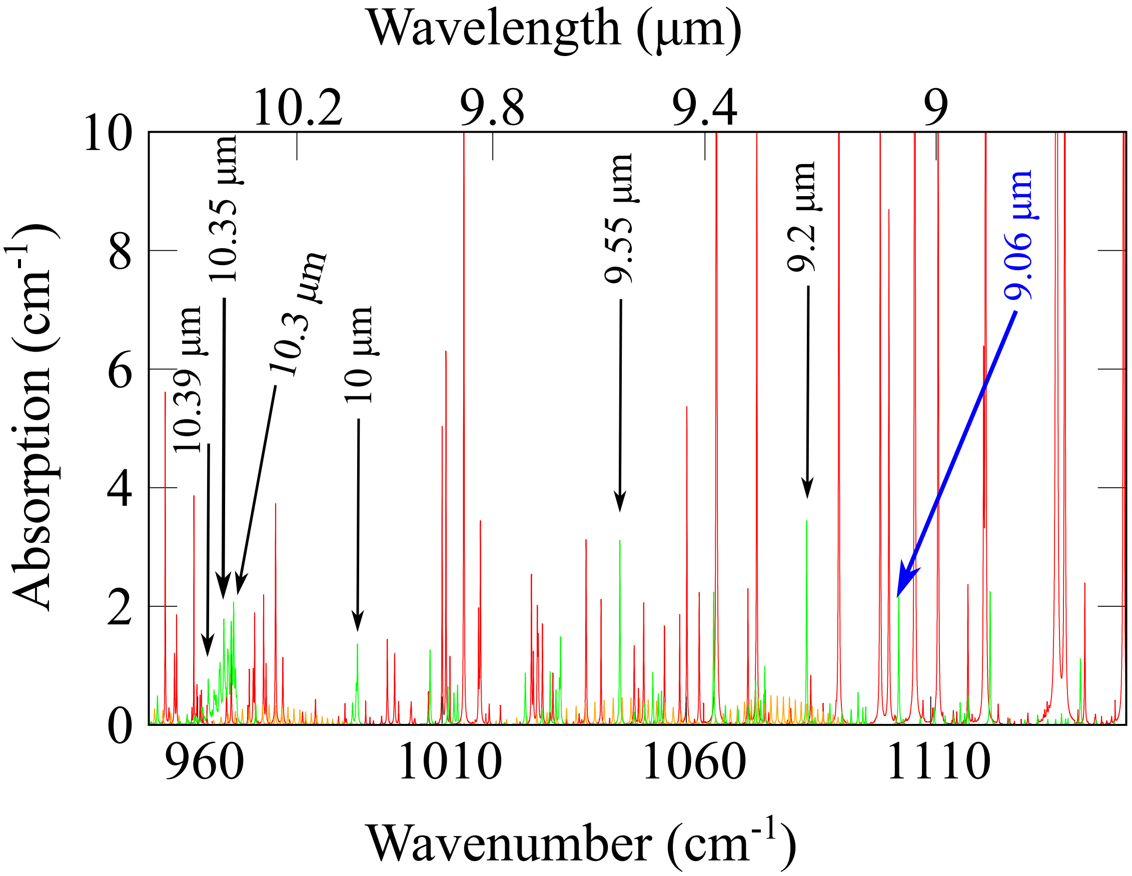

Figure S.1: **Simulated absorption spectra using parameters from HITRAN database[1]**. The gaseous matrix for which the simulations were performed includes 0.5 ppm of NH_3 (green curve), 30000 ppm of water (red curve), and 1000 ppm of CO_2 (orange curve) for $T = 333$ K, $P = 1$ atm in the spectral region from 950 to 1150 cm^{-1} (8.6-11.1 μm). Seven promising wavelenghts in this region has been previously investigated: 962.17 cm^{-1} (10.39 μm),[2] 965.35 cm^{-1} (10.35 μm),[3] 970.8 cm^{-1} (10.3 μm),[4] 993 cm^{-1} (10 μm),[5] 1046.4 cm^{-1} (9.55 μm),[6] 1085.825 cm^{-1} (9.2 μm)[7] and 1103.46 cm^{-1} (9 μm)[8–10] mainly for gas NH_3 monitoring at room temperature. Our specific selection at 1103.46 cm^{-1} (blue arrow) allows a strong ammonia absorption feature combined with a minimum in water vapor absorption and CO_2 interference in comparison to the other wavelengths (black arrows) in the same region.

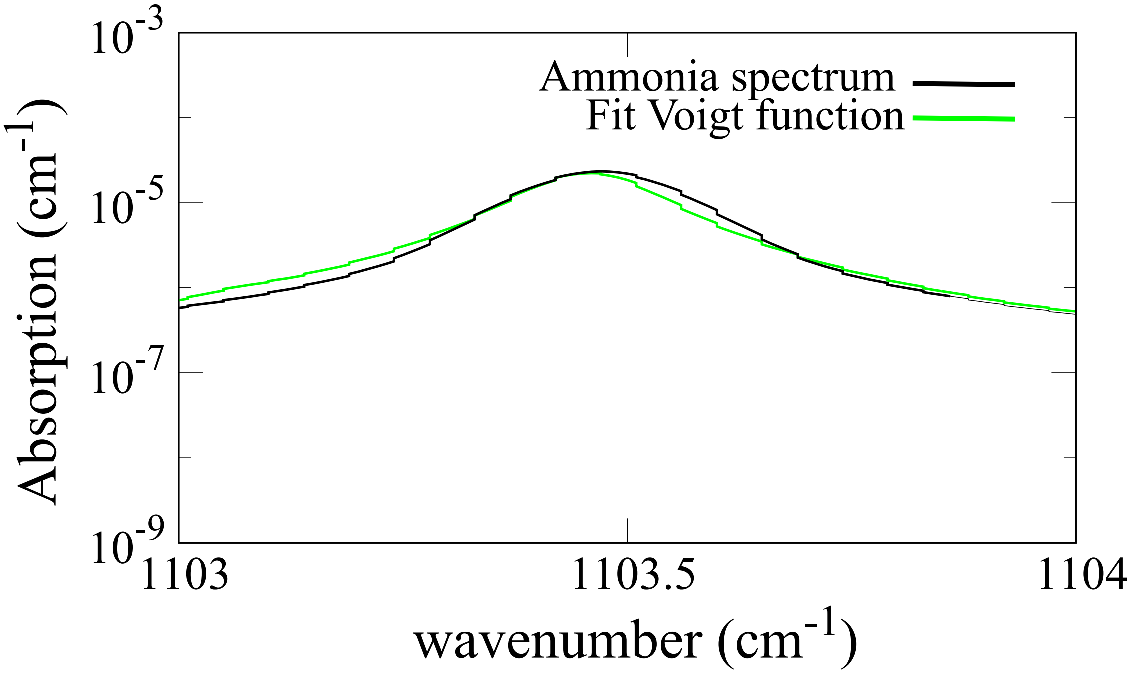

Figure S.2: **The peak of the ammonia absorption band is fitted by the pseudo-Voigt function compared to the simulated spectrum from the HITRAN database.** The spectra in Figure S.1 correspond to simulated Voigt profiles on the HITRAN database considering the environmental parameters and the contour parameters of the absorption line profiles. To facilitate similar spectroscopic simulations, the absorption band should be well approximated by a Voigt-like function $V_g(x)$,[11] for example, a linear combination of Lorentzian, $L(x)$, and Gaussian $G(x)$, $V_g(x) = y_0 + A[m \times L(x) + (1-m) \times G(x)]$, where $L = 2w/[4\pi(x-x_c)^2 + \pi w^2]$ and $G(x) = \sqrt{4\ln 2}/(\sqrt{\pi}w)\exp[-4\ln 2(x-x_c)^2/w^2]$. The fitting parameters were found using the Levenberg-Marquardt algorithm:[12,13] $y_0 = 1 \times 10^{-7}$ cm^{-1}, $A = 6.0344 \times 10^{-6}$, $m = 0.5943$, $w = 0.1961$ cm^{-1}, and $x_c = 1103.47$ cm^{-1}.

Section S2

Here, we present a set of figures (S.3-5) related to the processes involved in evaluating the performance of the gas sensor (multiSense module) response, along with corresponding data.

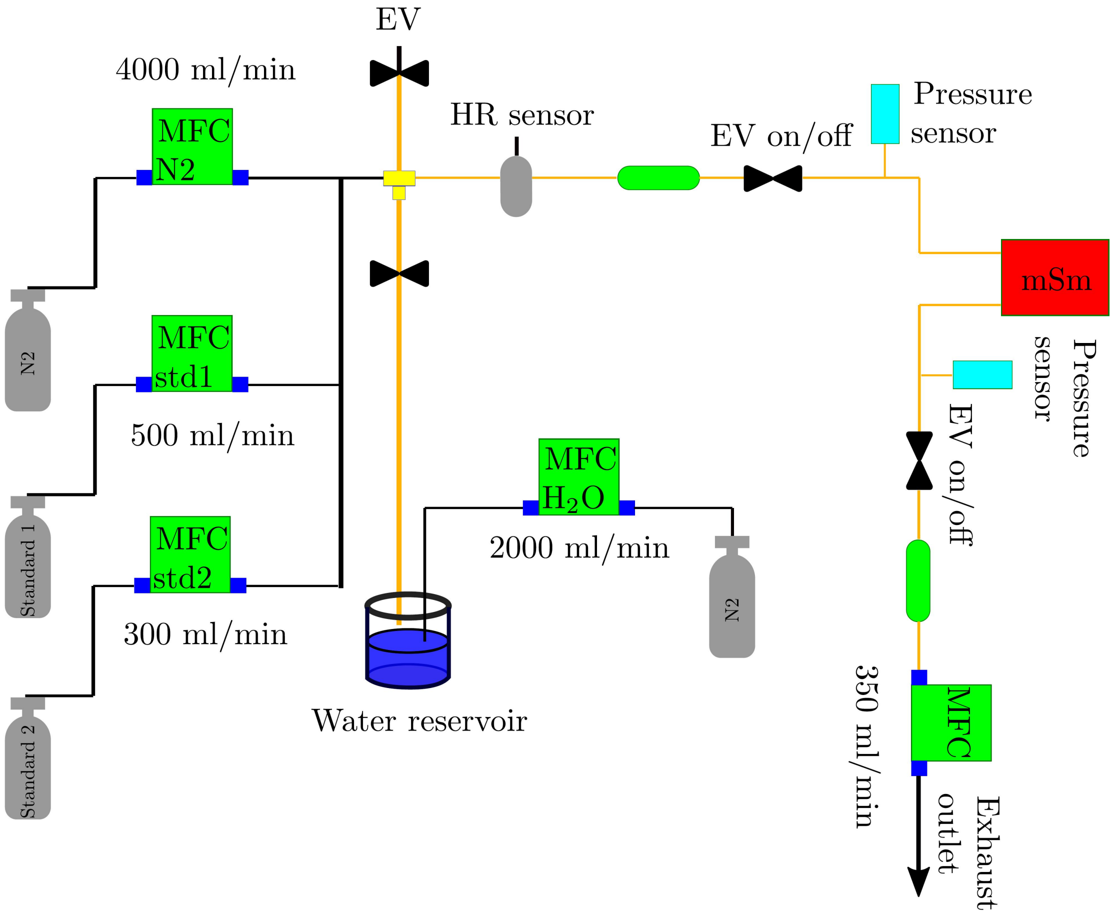

Figure S.3: **Simplified diagram of the laboratory test bench to evaluate the performance of the gas sensor response, i.e. multiSense module (mSm)**. Different ammonia (NH_3) concentrations were sent to mSm by controlling the flow of the reference gas mixture by means of mass flow controllers (MFC) (Bronkhorst, Germany), which sets the final flow rate at 350 ml/min. This setup allows us to prepare various gas mixtures of high precision, providing several dilutions of NH_3 and nitrogen (N_2) calibrated mixtures. The pre-diluted test-gas is specified by analyte concentration of 500 ppm NH_3 within a pressurized cylinder, whereas the N_2 mixture is introduced into the multiSense module after passing through humidifier stabilizing the temperature and the gas flow. All measurements are conducted under atmospheric pressure conditions, while the pressure itself is continuously monitored using pressure sensors.

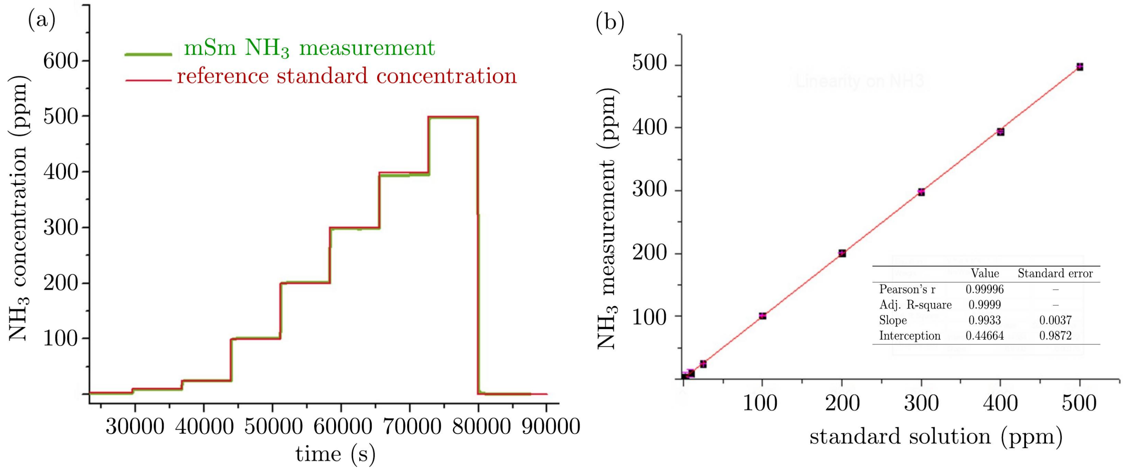

Figure S.4: **Assessing the performance of the mSm in measuring gaseous ammonia.** (a) A exhaustive data acquisition spanning 16 hours was conducted to evaluate the module's linear response across the ammonia concentration range 0 to 500 ppm. (b) The linear relationship between the sensor's response and the concentration of the standard solution samples. The inset contains a table of statistical information related to the linear response of the mSm. The mSm was securely housed within the analyzer rack to assess its thermal performance, electronic noise, and vibrations. A measured LOD of 0.18ppm in 60 s averaging was obtained. Additionally, a resolution below 0.1 ppm was demonstrated for gas phase measurements using the mSm.

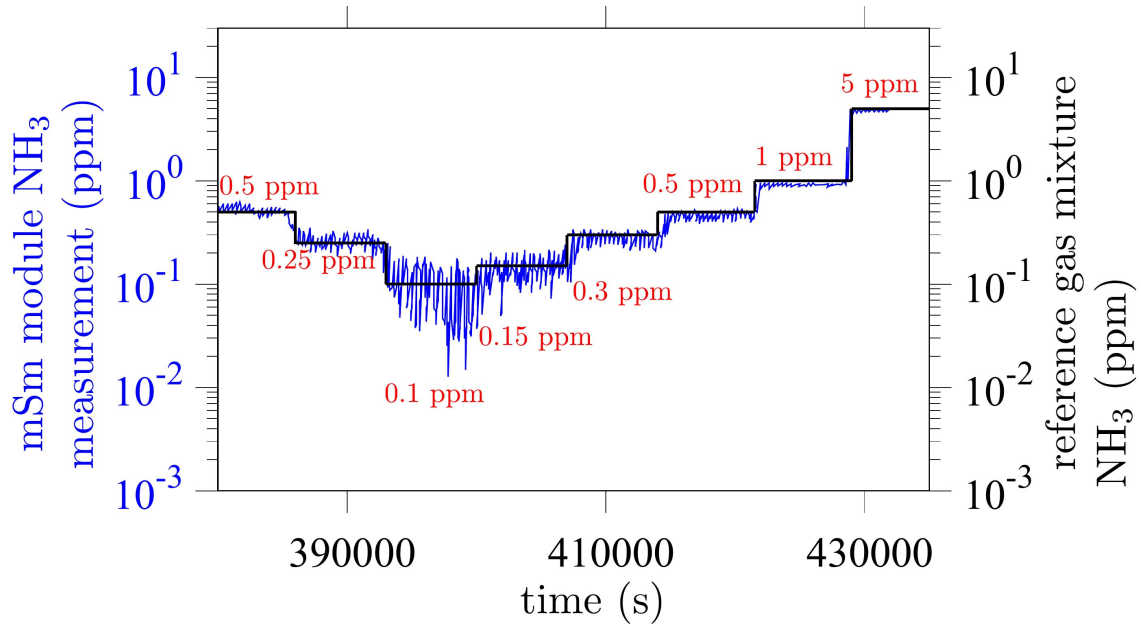

Figure S.5: **Demonstrating the mS module's capability to measure traces of gaseous ammonia near the limit of detection.** The measurements are reproducible, and no hysteresis was observed. Each concentration step is maintained for two hours. This graph also illustrates a linear measurement range between 0 and 5 ppm of NH_3.

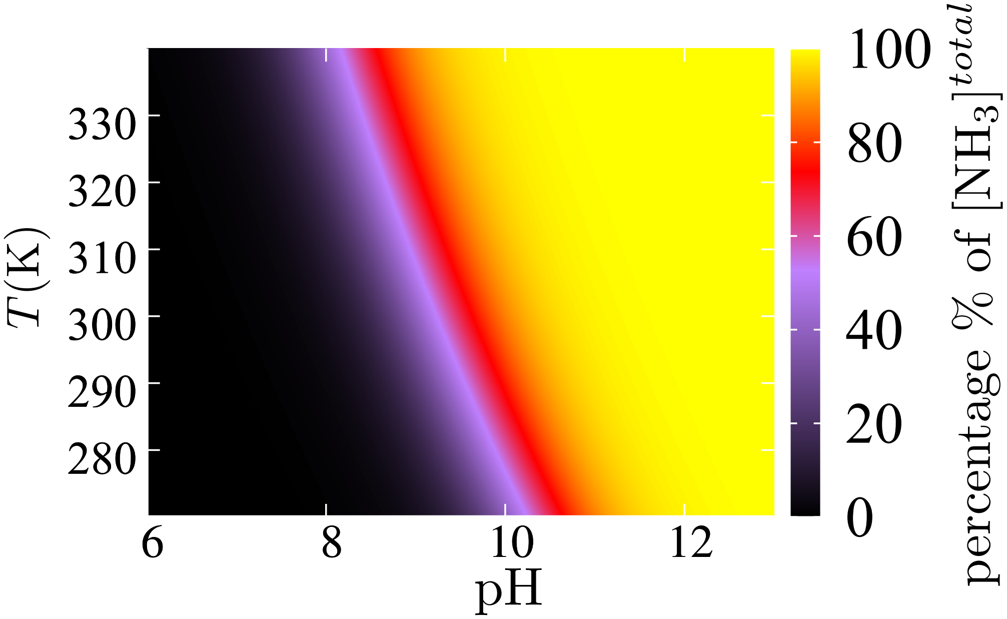

Figure S.6: Colormap illustrating the dependence of free ammonia percentage [Eq. (S.8)] on pH and temperature.

Section S3

In this section, we provide a more detailed description of the ammonia stripping method employed to extract gaseous ammonia from the water samples. The ammonia stripping process employs a mass transfer principle.[14,15] In this approach, water comes into contact with air, facilitating the removal of ammonia gas from the water. Ammonia in water exists in two forms: ammonium ions (NH_4^+) and ammonia gas (NH_3). The proportions of ammonia gas and ammonium ions are immediately affected by the water's pH and temperature. Ammonia nitrogen within water exists in a state of equilibrium between its molecular and ionic forms, as depicted by the following reaction

$$NH_3 + H_2O \leftrightarrow NH_4^+ + OH^-, \tag{S.1}$$

while the dissociation of water is described by the equilibrium reaction

$$H_2O \leftrightarrow H_3O^+ + OH^-. \tag{S.2}$$

The ammonia fraction, denoted as $f_{\mathrm{NH_3}}$, governs the concentration ratio of free ammonia $[\mathrm{NH_3}]$ and total ammonia $[\mathrm{NH_3}]^{total} = [\mathrm{NH_3}] + [\mathrm{NH_4^+}]$. It can also be defined as

$$f_{\mathrm{NH_3}} = \frac{1}{1 + \frac{K_{\mathrm{NH_3}}}{K_{\mathrm{H_20}}} \times 10^{-\mathrm{pH}}}, \tag{S.3}$$

where

$$K_{\mathrm{H_20}} = [\mathrm{H_3O^+}][\mathrm{OH^-}] \tag{S.4}$$

and

$$K_{\mathrm{NH_3}} = \frac{[\mathrm{NH_4^+}][\mathrm{OH^-}]}{[\mathrm{NH_3}]} \tag{S.5}$$

determine the ionization constants of water and ammonia respectively. The ionization constants[16,17] and the ammonia fraction[18] have been calculated semi-empirically as

$$\begin{aligned} \mathrm{p}K_{\mathrm{NH_3}} = 4 \cdot 10^{-8} \cdot T^3(\mathrm{K}) + 9 \cdot 10^{-5} \cdot T^2(\mathrm{K}) \\ -0.0356 \cdot T(K) + 10.072, \end{aligned} \tag{S.6}$$

$$\begin{aligned} \mathrm{p}K_{\mathrm{H_2O}} = 24691.6 \cdot T^{-1}(\mathrm{K}) + 405.867 \cdot \log(T(\mathrm{K})) \\ - 0.488054 \cdot T(\mathrm{K}) \\ + 2.37842 \cdot 10^{-4} \cdot T^2(\mathrm{K}) - 948.73, \end{aligned} \tag{S.7}$$

and

$$f_{\mathrm{NH_3}} = \left(1 + \frac{10^{-\mathrm{pH}}}{10^{-\left(0.09018 + \frac{2729.92}{T(\mathrm{K})}\right)}}\right)^{-1}, \tag{S.8}$$

where $T(\mathrm{K})$ is temperature in Kelvin. Typical stripping processes require a sample temperature between 293 and 323 K, whereas pH values range between 10 and 12. This stems from the dependence of free ammonia on pH and temperature as demonstrated in Figure S.6. Hence, the requirement for a reagent in the form of a basic solution to control pH levels. In

our experiment, we utilized a sodium hydroxide (NaOH) solution, leading to a substantial alkaline pH of approximately 12 and, consequently, an ammonia fraction nearing unity; see the yellow shaded region in Figure S.6. The ammonia stripping/sampling compartment of the analyzer was structured as shown in Figure 1a. The stripping column was located at the center of the rack and with a working volume of 0.5 L. We note that the height of the reservoir (stripping pot) has been designed to be 20 cm to avoid water droplets reaching the top, where the air sampling port is located. A peristaltic sampling pump was installed above to ensure a proper filling of the stripping column with liquid sample, whereas a peristaltic pump was introducing periodically 0.8 ml of the NaOH solution. Hereafter, an air pump was activated introducing air required for the ammonia removal, i.e. degassing NH_3 into the circulating air. The tail gas, a mixture of air and ammonia, was released through the top of the stripping column and then absorbed by the mSm unit.

References

(1) `https://hitran.org/`, Accessed: Oct. 3, 2023.

(2) Peng, W.; Sur, R.; Strand, C.; Spearrin, R.; Jeffries, J.; Hanson, R. High-sensitivity in situ QCLAS-based ammonia concentration sensor for high-temperature applications. *Applied Physics B* **2016**, *122*, 188.

(3) Li, B.; Feng, C.; Wu, H.; Jia, S.; Dong, L. Calibration-free mid-infrared exhaled breath sensor based on BF-QEPAS for real-time ammonia measurements at ppb level. *Sensors and Actuators B: Chemical* **2022**, *358*, 131510.

(4) Mičica, M.; Eliet, S.; Vanwolleghem, M.; Motiyenko, R.; Pienkina, A.; Margulès, L.; Postava, K.; Pištora, J.; Lampin, J.-F. High-resolution THz gain measurements in optically pumped ammonia. *Optics express* **2018**, *26*, 21242–21248.

(5) Tsai, T.; Wysocki, G. External-cavity quantum cascade lasers with fast wavelength scanning. *Applied Physics B* **2010**, *100*, 243–251.

(6) Milton Filho, B.; da Silva, M. G.; Sthel, M. S.; Schramm, D. U.; Vargas, H.; Miklós, A.; Hess, P. Ammonia detection by using quantum-cascade laser photoacoustic spectroscopy. *Applied optics* **2006**, *45*, 4966–4971.

(7) Silver, J. A.; Bomse, D. S.; Stanton, A. C. Diode laser measurements of trace concentrations of ammonia in an entrained-flow coal reactor. *Applied optics* **1991**, *30*, 1505–1511.

(8) Ji, Y.; Duan, K.; Lu, Z.; Ren, W. Mid-infrared absorption spectroscopic sensor for simultaneous and in-situ measurements of ammonia, water and temperature. *Sensors and Actuators B: Chemical* **2022**, *371*, 132574.

(9) Wang, R.; Peng, J.; Chen, J.; Ti, C.; Wang, G.; Liu, K.; Gao, X. Standoff sub-ppb level measurement of atmospheric ammonia with calibration-free wavelength modulation spectroscopy. *Spectrochimica Acta Part A: Molecular and Biomolecular Spectroscopy* **2023**, *286*, 121929.

(10) Miller, D.; Sun, K.; Tao, L.; Khan, M.; Zondlo, M. Open-path, quantum cascade laser-based sensor for high resolution atmospheric ammonia measurements. *Atmospheric Measurement Techniques Discussions* **2013**, *6*.

(11) Xia, J.; Zhu, F.; Kolomenskii, A. A.; Bounds, J.; Zhang, S.; Amani, M.; Fernyhough, L. J.; Schuessler, H. A. Sensitive acetone detection with a mid-IR interband cascade laser and wavelength modulation spectroscopy. *OSA Continuum* **2019**, *2*, 640–654.

(12) Moré, J. J. The Levenberg-Marquardt algorithm: implementation and theory. Numerical analysis: proceedings of the biennial Conference held at Dundee, June 28–July 1, 1977. 2006; pp 105–116.

(13) Apostolakis, A.; Pereira, M. F. Controlling the harmonic conversion efficiency in semiconductor superlattices by interface roughness design. *AIP Advances* **2019**, *9*.

(14) Matter-Müller, C.; Gujer, W.; Giger, W. Transfer of volatile substances from water to the atmosphere. *Water Research* **1981**, *15*, 1271–1279.

(15) Kim, E. J.; Kim, H.; Lee, E. Influence of ammonia stripping parameters on the efficiency

and mass transfer rate of ammonia removal. *Applied sciences* **2021**, *11*, 441.

(16) Olofsson, G.; Hepler, L. G. Thermodynamics of ionization of water over wide ranges of temperature and pressure. *Journal of Solution Chemistry* **1975**, *4*, 127–143.

(17) Bonmatí, A.; Flotats, X. Air stripping of ammonia from pig slurry: characterisation and feasibility as a pre-or post-treatment to mesophilic anaerobic digestion. *Waste management* **2003**, *23*, 261–272.

(18) Hansen, K. H.; Angelidaki, I.; Ahring, B. K. Anaerobic digestion of swine manure: inhibition by ammonia. *Water research* **1998**, *32*, 5–12.
