# Supplementary figures and images for: Photoacoustic Spectroscopy Using a Quantum Cascade Laser for Analysis of Ammonia in Water Solutions
Source: ACS Omega. 2024 Apr 17;9(17):19127–35. doi: 10.1021/acsomega.3c10175 (PMC11064027; doi:10.1021/acsomega.3c10175)

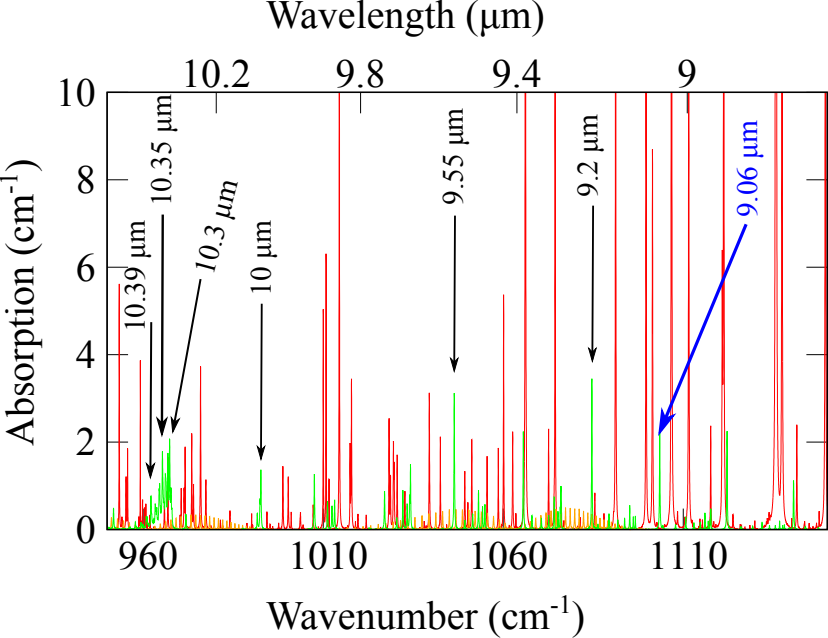

Supplement: Supplementary file 2 — ao3c10175_si_002.zip [file ao3c10175_si_002.zip › figS1.pdf]

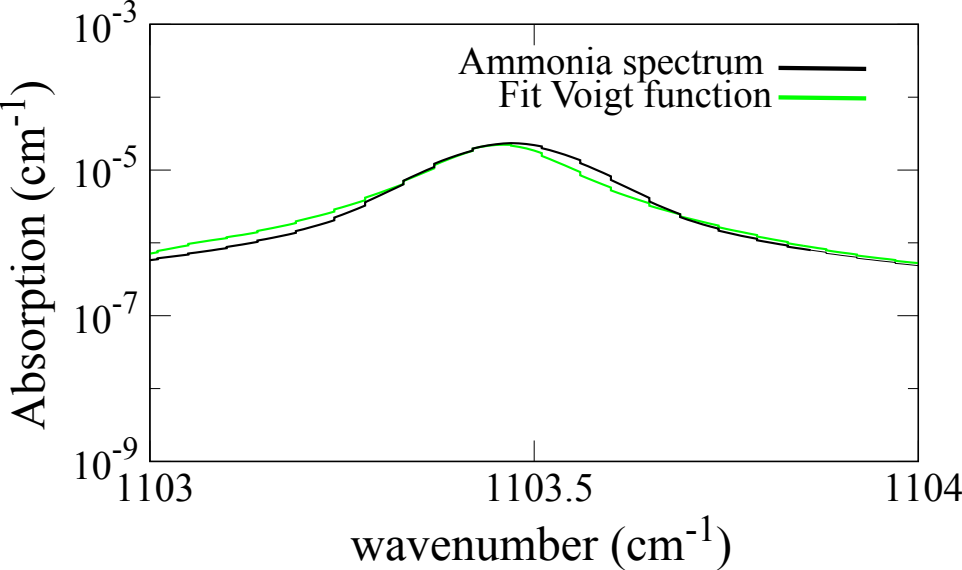

Supplement: Supplementary file 2 — ao3c10175_si_002.zip [file ao3c10175_si_002.zip › figS2.pdf]

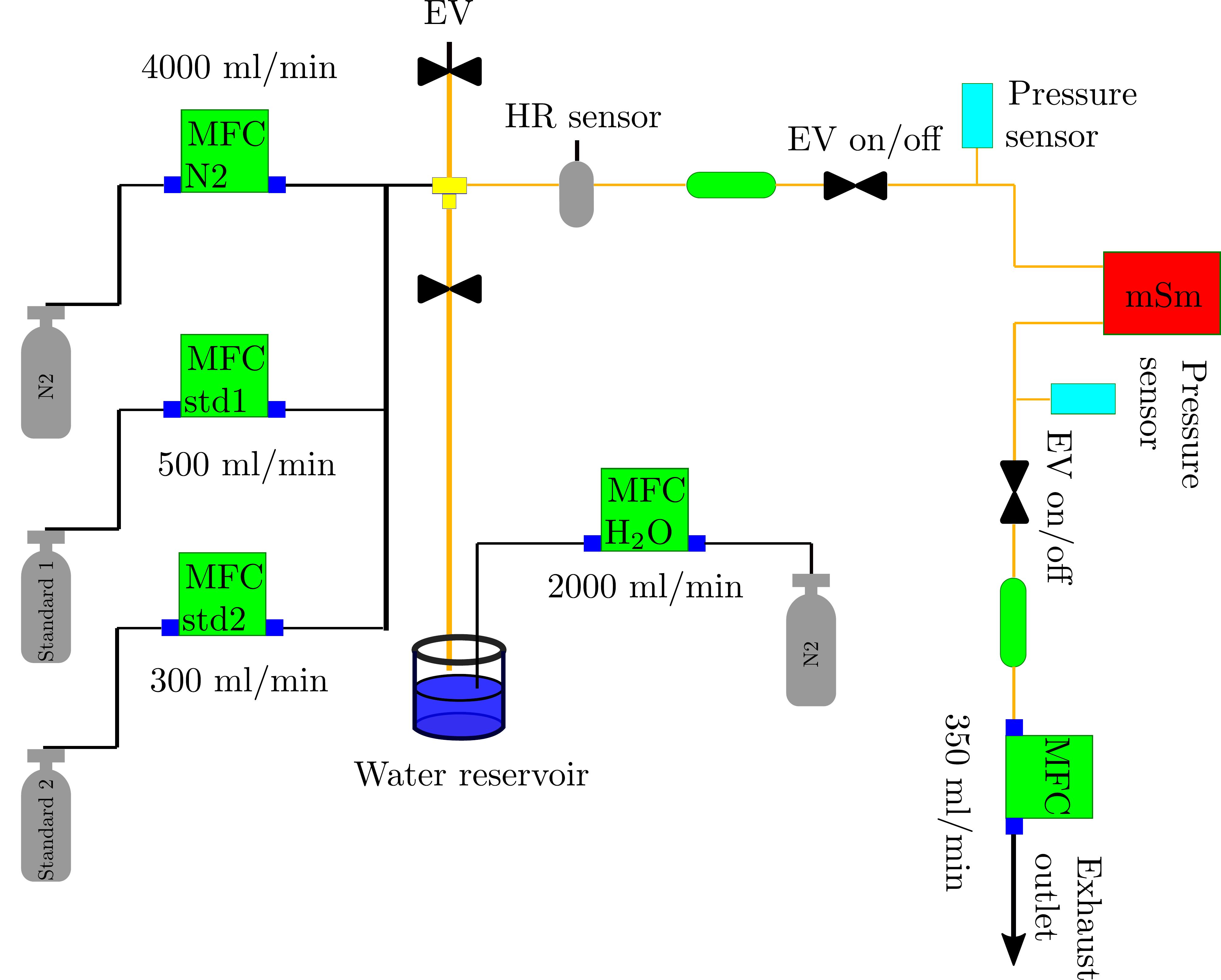

Supplement: Supplementary file 2 — ao3c10175_si_002.zip [file ao3c10175_si_002.zip › figS3.jpg]

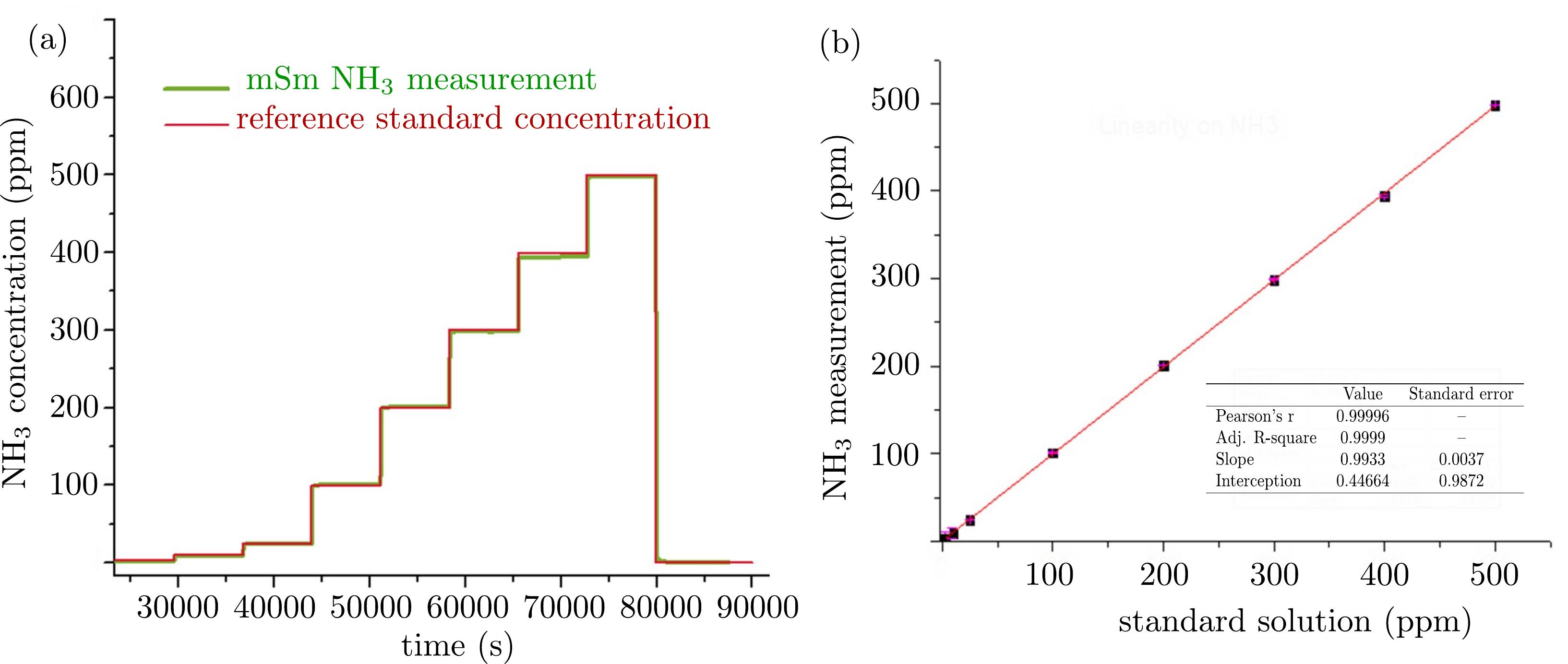

Supplement: Supplementary file 2 — ao3c10175_si_002.zip [file ao3c10175_si_002.zip › figS4.jpg]

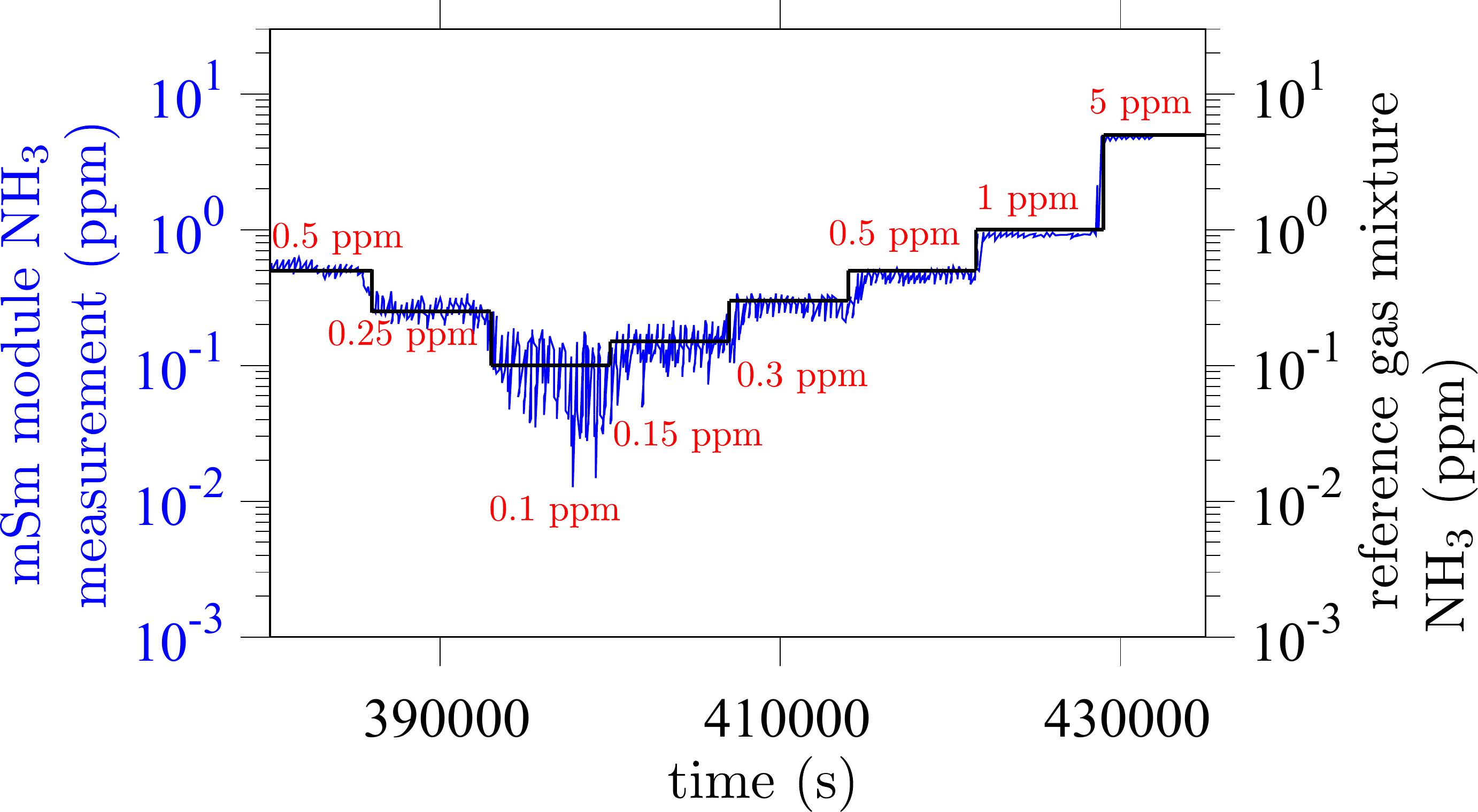

Supplement: Supplementary file 2 — ao3c10175_si_002.zip [file ao3c10175_si_002.zip › figS5.jpg]

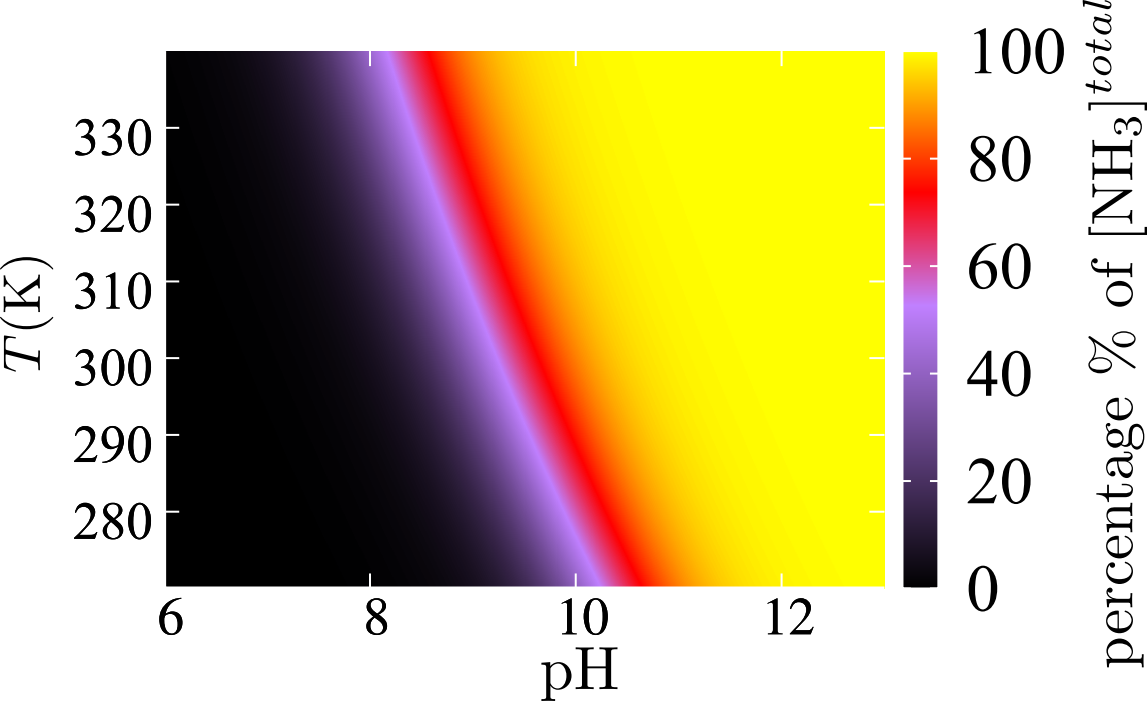

Supplement: Supplementary file 2 — ao3c10175_si_002.zip [file ao3c10175_si_002.zip › figS6.pdf]
